# A Surprising Source of Self-Motivation: Prior Competence Frustration Strengthens One’s Motivation to Win in Another Competence-Supportive Activity

**DOI:** 10.3389/fnhum.2018.00314

**Published:** 2018-08-03

**Authors:** Hui Fang, Bin He, Huijian Fu, Huijun Zhang, Zan Mo, Liang Meng

**Affiliations:** ^1^School of Management, Guangdong University of Technology, Guangzhou, China; ^2^Laboratory of Neuromanagement and Decision Neuroscience, Guangdong University of Technology, Guangzhou, China; ^3^School of Business and Management, Shanghai International Studies University, Shanghai, China; ^4^Laboratory of Applied Brain and Cognitive Sciences, Shanghai International Studies University, Shanghai, China; ^5^Center for Functional Neuroimaging, Department of Neurology, University of Pennsylvania, Philadelphia, PA, United States

**Keywords:** competence, competence frustration, intrinsic motivation, need restoration, self-determination theory, event-related potentials, feedback-related negativity

## Abstract

According to self-determination theory (SDT), competence is among the three basic psychological needs essential for one’s well-being and optimal functioning, and the frustration of these needs is theoretically predicted to induce a restorative response. While previous studies have explored the restoration process of autonomy and relatedness, empirical evidence for such a process is still lacking for competence. In order to explore this process and to examine the effect of prior competence frustration on one’s motivation to win in a subsequent competence-supportive task, we adopted a between-group experimental design and manipulated one’s competence frustration through task difficulty in an electrophysiological study. Participants in both groups were instructed to work on the time-estimation task and the stop-watch task in two successive sessions respectively. Participants in the experimental group were asked to complete a highly difficult task in the first session and a task of medium difficulty in the second session, while those in the control group were instructed to work on tasks of medium difficulty in both sessions. In the second session, an enlarged feedback-related negativity (FRN) loss-win difference wave (d-FRN) was observed in the experimental group compared to the control group, indicating that the competence-frustrated participants have an enhanced motivation to win in a subsequent competence-supportive task. Thus, results of the present study provided original neural evidence for the restoration process of frustrated competence, which provided important guidelines for the managerial practice.

## Introduction

In our daily life, we frequently observe the phenomenon that, instead of being devastated, lots of people will seek an opportunity to prove themselves after a setback. For instance, people who failed an interview may regain confidence and happiness by managing to succeed in other domains (i.e., to win a tennis match). Their behaviors may seem irrational at first glance. After all, winning a tennis match itself would not help them change the interview result. However, this act helps people to restore their undermined perceived competence. This vivid scenario shows that a need restoration process of competence may exist and that individuals may actively enforce self-regulation to fulfill their basic psychological needs.

In order to clarify and integrate varied influencing factors of motivation, psychology and management researchers proposed multiple theories. Among them, self-determination theory (SDT) has emerged as one of the most influential and well-established frameworks of motivation. One major contribution of SDT is that it conceptualizes the three basic psychological needs of autonomy, competence and relatedness as essential and innate for one’s psychological growth, internalization and well-being (Deci and Ryan, 2000; Reis et al., 2000). Autonomy reflects one’s need to act with a sense of discretion of his/her own behaviors and to feel psychologically self-directed, while relatedness refers to the need to interpersonally connect with others, to give affection, and to receive love and care in return. Finally, competence is defined as the need to feel effective and mastery, and to demonstrate and improve one’s abilities (Deci and Ryan, 2000). As a fundamental basic psychological need, the importance of competence satisfaction has been explored in a multitude of fields, such as education, work, health and sports (Milyavskaya and Koestner, 2011). It is widely reported that competence satisfaction is positively correlated with work motivation, job satisfaction, life satisfaction as well as the general well-being (Van den Broeck et al., 2016).

Besides exploring the positive effects of competence satisfaction, recent studies have begun to examine the negative effects of competence frustration. Competence frustration refers to feelings of failure or inadequacy, and doubt over one’s own abilities (Bartholomew et al., 2011). When challenges are set too high, negative feedback is provided, and/or the sense of mastery gets undermined by targeted criticism and social comparisons, people would experience competence frustration (Ryan and Deci, 2017). Studies showed that competence frustration is often accompanied by negative outcomes, such as ill-being (Bartholomew et al., 2014), job burnout (Gillet et al., 2015b), counter-productive work behavior (Van den Broeck et al., 2014), cynicism and turnover intentions (Gillet et al., 2015a), disengagement (Jang et al., 2016) and undermined intrinsic motivation (Fang et al., 2017).

Given that experiencing competence satisfaction is crucial to optimal functioning, it is hard to believe that people would passively accept competence frustration without making any defensive reactions. Indeed, previous studies have demonstrated that the frustration of basic psychological needs would lead to a restoration process (Fiske, 2004; Veltkamp et al., 2009). In a recent experimental study, autonomy-frustrated participants were found to pay more attention to autonomy-related stimuli in a subsequent task, which would help them restore undermined autonomy (Radel et al., 2011). Moreover, individuals who experienced autonomy frustration were found to have a greater intrinsic motivation in a subsequent task if this new task gives them a glimpse of autonomy satisfaction (Radel et al., 2014). Besides autonomy, previous studies also reported that individuals who experienced relatedness frustration tried harder and performed better in the next task if this task provided them the opportunity to feel socially accepted (DeWall et al., 2008). However, once people experienced competence frustration, whether they would take actions to restore their perceived competence and be more eager to win in a subsequent competence-supportive task remains elusive. Thus, the aim of this study is to explore the restoration process of competence and to establish the causal relationship between prior competence frustration and one’s motivation to win in another activity.

In this experimental study, we adopted a between-subject design. Participants in both groups were instructed to attend two sessions, and they worked on the same task both in session 1 (the time-estimation task, TE) and session 2 (the stop-watch task, SW). In session 1, competence frustration was manipulated by setting different difficulties for the same task, which has been suggested to be a both simple and effective means of competence frustration manipulation (Ryan and Deci, 2017). In session 2, all participants worked on another task of medium difficulty, which was found to be competence-supportive to a great extent (Meng et al., 2016; Ma et al., 2017). In order to examine the effect of prior competence frustration on one’ motivation to win in a subsequent competence-supportive task, electrophysiological data of all participants were recorded and analyzed. Specifically, we resorted to feedback-related negativity (FRN), a representative event-related potentials (ERPs) component observed during feedback processing and outcome evaluation to measure one’ motivation level (Ma et al., 2014a; Meng and Ma, 2015).

As a negative deflection, FRN generally peaks between 250 ms and 350 ms after feedback onset and is concentrated over the fronto-central electrodes (for a recent literature review, see San Martín, 2012). Source localization studies have demonstrated that the neural generator of the FRN lies in the anterior cingulate cortex (Müller et al., 2005; Bocquillon et al., 2014; Hauser et al., 2014). In order to illustrate the cognitive meaning of FRN, scholars have proposed and developed two mainstream theories, which are reinforcement learning theory and motivational significance theory. According to reinforcement learning theory, FRN is sensitive to the valence of outcome feedback, being more pronounced for negative feedback than for the positive one. The increased FRN amplitude elicited by negative outcomes is resulted from the decreased dopaminergic activity when observing events worse than expected (Holroyd and Coles, 2002). We predicted to replicate the valence effect in this study.

While reinforcement learning theory is helpful in explaining the valence effect of FRN, motivational significance theory takes a difference wave approach and argues that FRN loss-win difference wave (d-FRN) represents the subjective evaluation of the motivational impact of outcome events (Gehring and Willoughby, 2002; Yeung et al., 2005; Masaki et al., 2006). Previous studies have consistently suggested that the d-FRN amplitude reflects the motivational significance of outcomes in both gambling tasks (Masaki et al., 2006; Zhou et al., 2010; Ma et al., 2011) and effort-requiring tasks (Ma et al., 2014b; Meng and Ma, 2015). To be specific, when outcomes in a given experimental condition bear more motivational significance to participants, an enhanced d-FRN would be observed upon feedback (Yeung et al., 2005; Fukushima and Hiraki, 2009; San Martín, 2012; Meng and Ma, 2015). As we hypothesized that individuals who experienced competence frustration beforehand may actively seek to restore their perceived competence in a subsequent less-demanding task, we predicted that they would have a more sustained motivation to win in another competence-supportive task, resulting in a significantly more pronounced d-FRN upon feedback.

It is worth pointing out that, in this study we resort to the d-FRN to measure one’s motivation to win rather than intrinsic motivation. Specifically, as we aim to provide direct empirical evidences for the competence restoration process, we examine the effect of prior competence frustration on one’s motivation to win (as reflected in the magnitude of d-FRN) in another competence-supportive activity. In previous studies, researchers suggested the d-FRN upon feedback as an electrophysiological indicator of intrinsic motivation (Ma et al., 2014a; Meng and Ma, 2015) either when external rewards are not provided or when monetary incentives are irrelevant to task performances. In this study, as we did not collect subjective ratings on intrinsic motivation from the participants, we cannot be conclusive that the d-FRN reflects intrinsic motivation here. Thus, intrinsic motivation would only be briefly discussed as a possible explanation of the observed d-FRN effect in the “DISCUSSION” section of this article.

Besides establishing a causal link between competence frustration in a prior activity and one’s motivation to win in the subsequent competence-supportive one, another aim of this study is to explore effects of personality traits on one’ motivation to win during the need restoration process. One personality trait that attracts our attention is achievement goal orientation, which refers to one’s beliefs towards the goals they form to succeed, and the driving forces of their learning behaviors (Ames, 1992; Pintrich, 2000; Kaplan and Maehr, 2007). There are two distinct achievement goal orientations. While mastery goal concerns the development of competence and task mastery, performance goal pays attention to the demonstration of one’s competence to other people. Each individual has the two orientations at the same time. However, they may vary in levels. Thus, one can be classified as either more mastery-orientated or more performance-orientated. Previous studies consistently reported an undermining effect of performance goal orientation on one’s intrinsic motivation (Ryan et al., 1991; Kaplan and Maehr, 2007; Lee, 2010; Barić et al., 2014). Extending this line of studies, in this study we explored whether mastery versus performance goal orientation would affect one’s motivation to win (as reflected in the magnitude of d-FRN) in a subsequent less demanding activity after experiencing competence frustration.

## Materials and Methods

### Participants

Forty-eight healthy, right-handed participants took part in this study, ranging in age from 19 years to 24 years (*M* = 19.50, SD = 0.93). A power analysis was conducted to determine the sample size before we started this experiment. We assumed the effect size (f) to be 0.4 and the error probability (α) to be 0.05. The suggested sample size is 44. Thus, our sample size meets the requirement. All participants were randomly assigned to either the control (*N* = 24, 14 males) or the experimental group (*N* = 24, 12 males). All participants were registered students from Guangdong University of Technology. They had normal vision after correction and no history of neurological disorders or mental diseases. The study was approved by the Internal Review Board of School of Management, Guangdong University of Technology. All participants provided written informed consent before the experiment formally started.

### Stimuli and Procedure

**Figure 1:**
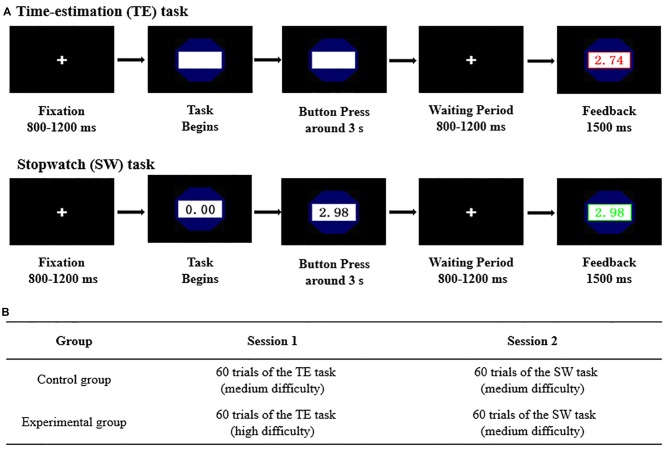
The experimental paradigm. **(A)** Illustration of time-estimation (TE) and stopwatch (SW) tasks. **(B)** Demonstration of the experimental procedure.

Subjects were comfortably seated in a dimly lit, sound-attenuated and electrically shielded room. Experimental stimuli were presented at the center of a computer screen at a distance of 100 cm, with a visual angle of 6.2° × 5.4°. Subjects were instructed to use a keypad to complete tasks all along. The experiment consisted of two sessions, each containing 60 trials. As illustrated in Figure 1B, participants in both groups were instructed to work on the TE task in session 1 and the SW task in session 2, respectively.

In session 1, participants were instructed to accomplish the TE task and to estimate durations of around 3 s. After TE began, participants should respond by pressing any button on the keypad if they considered that the elapsed time was close to 3 s (Meng and Ma, 2015). The closer, the better. In order to manipulate competence frustration between groups in session 1, participants in the control group were instructed to work on a TE task of medium difficulty (the success interval of which is [2.75 s, 3.25 s]), while those in the experimental group were assigned an overwhelmingly difficult TE task (the success interval of which is [2.93 s, 3.07 s]). In session 2, participants from both groups were instructed to complete the same SW task of moderate difficulty (the success interval of which is [2.93 s, 3.07 s]), which is competence-supportive. During the SW game, a SW would automatically start, and participants should try their best to stop the watch around 3 s (Murayama et al., 2010; Albrecht et al., 2014; Ma et al., 2014a, 2017; Meng et al., 2016). Again, the closer, the better. All these time windows were determined by a pilot study conducted before the formal experiment, which ensured that typical participants would succeed in around 15% and 50% trials when working on the overwhelmingly difficult task and the task of medium difficulty respectively. It is worth pointing out that in order to prevent any confounds, participants were only told that the whole experiment would be divided by two sessions, and they were introduced the specific task immediately before the corresponding session began.

As demonstrated in Figure 1A, each trial commenced with a cross symbol that lasted for 800–1200 ms. After the task began, participants may press any button they like on the keypad to complete the task. Following the button press, a fixation period that lasted for 800–1200 ms was demonstrated. By the end of a trial, participants would receive their performance feedback for 1500 ms. If the response was close enough to the target, which fell into the pre-determined interval, task performances would be displayed in a green font and with a green border. However, if the behavioral response occurred outside of the pre-defined success interval, task performances would be displayed in a red font corresponding with the red border instead. There was a randomized blank interval that lasted for 600–1000 ms before the next trial started.

All subjects were required to complete an online questionnaire through a professional survey website before the experiment was implemented. The scale developed by Button was adopted to evaluate the achievement goal orientation of candidate participants (Button et al., 1996). The questionnaire on achievement goal orientation is included in Supplementary Table S2. Odd number items measure one’s performance goal orientation, while even number items measure one’s mastery goal orientation. The Cronbach’s α of the sub-scales for performance goal orientation and mastery goal orientation are 0.692 and 0.771, respectively. At the end of the experiment, participants were asked to rate their competence frustration level when working on the TE task. We measured one’ perception of competence frustration by adapting the basic psychological need satisfaction and frustration scale—work domain (Chen et al., 2015; Schultz et al., 2015), which is shown in Supplementary Table S1. The Cronbach’s α of this scale is 0.815. It is worth pointing out that, for all these scales, participants were asked to rate on a 7-point scale ranging from 1 (Do not fully agree) to 7 (Totally agree). Before the experiment formally started, subjects were told that they would receive ¥40 as compensation for their participation. Thus, their task performances were irrelevant to the final payments. To familiarize them with the tasks, a practice session adopting the formal task was implemented before the start of each session. After the experiment, subjects were debriefed and paid. Stimuli, recording triggers and response data were presented and recorded by E-Prime 2.0 (Psychology Software Tools, Pittsburgh, PA, USA).

### EEG Data Recordings and Analyses

The electroencephalogram (EEG) was recorded with the eego amplifier, using a Waveguard EEG Cap with 64 Ag/AgCl electrodes mounted according to the extended international 10–20 system (both manufactured by ANT Neuro, Enschede, Netherlands). Channel data were online band-pass-filtered from 0.1 Hz to 100 Hz and recorded at a sampling rate of 500 Hz. The left mastoid served as the on-line reference, and the EEG was off-line re-referenced to the mathematically averaged mastoids. Impedances were kept below 10 kΩ throughout the experiment. During off-line data analyses, EEG data were pre-processed adopting ASALab 4.10.1 (ANT Neuro, Enschede, Netherlands). Ocular artifacts were identified and corrected with the eye movement correction algorithm embedded in the ASALab program. The EEGs went through a digital low-pass filter at 30 Hz (24 dB/octave). For the FRN, time windows of 200 ms before and 800 ms after onset of the feedback were segmented, with the activity from −200 ms to 0 ms serving as the baseline. For each participant, the recorded EEGs over each recording site were averaged across each experimental condition. Trials containing amplifier clipping, bursts of electromyography activity, or peak-to-peak deflection that exceeded ±100 μV were excluded from the final averaging.

In this study, we decide to focus our analysis on a specific electrode cluster. While a pre-selection of electrodes might be reductionist, which does not provide much information on possible spatial differences during cognitive processing, this is a common practice for ERP studies, especially for those that focused on well-studied ERP components such as the FRN. As has been discussed in the introduction, the FRN is a negative deflection observed primarily at the fronto-central electrodes, which generally reaches its maximum magnitude around 300 ms after feedback onset (Nieuwenhuis et al., 2004; Torres et al., 2013). In most of the previous studies, FRN was measured at FCz (Oemisch et al., 2017; Fernandes et al., 2018), Fz and FCz (Megías et al., 2018), Fz, FCz and Cz (Hird et al., 2017), or Fz, Cz and Pz (Cohen et al., 2007). It was generally quantified as the mean amplitude in a chosen time window (Cohen et al., 2007; Hird et al., 2017; Fernandes et al., 2018; Megías et al., 2018). Recently, as one of the most famous EEG experts, Luck suggested that for classical ERP components such as the FRN, including electrode as a factor does not provide much useful information while may hide some significant results (Luck and Gaspelin, 2017). Following this suggestion, we selected an electrode cluster (FC1, FCz, FC2) for the FRN analysis based on grand averaged waveforms and its anterior distribution in this study. As the most negative peak of the FRN appeared around 245 ms after feedback onset, we used the mean amplitudes in the time window of 210–280 ms following feedback onset in a 2 (group) × 2 (outcome) repeated measure ANOVAs. The Greenhouse-Geisser correction for repeated measures was applied when necessary. While it might be interesting to test a possible mediation effect of competence frustration between our experimental manipulation of task difficulty and the d-FRN amplitude, this test was not conducted in this study. A major reason is that a typical mediation analysis requires a minimum number of participants, while most electrophysiological studies (including this one) fail to satisfy this requirement. An independent *t*-test was adopted in behavioral analyses. Specifically, to compare the mean error between the two groups, the mean absolute deviation around the central point (3 s) was calculated.

## Results

### Behavioral Results

The independent sample *t*-test showed that there was a significant difference in success rates (*M*_experimental_ = 0.16 (SD = 0.055), *M*_control_ = 0.55 (SD = 0.147); *t*_(46)_= 12.431, *p* < 0.001, cohen’d = 3.51) in the TE task during session 1. In addition, the level of competence frustration was significantly different between the control group and the experimental group (*M*_experimental_ = 4.625 (SD = 0.944), *M*_control_ = 3.052 (SD = 1.249); *t*_(46)_= −4.922, *p* < 0.001, cohen’d = 1.42), which confirmed that our manipulation was successful. For performance in the SW task during session 2, there were no significant differences in success rates (*M*_experimental_ = 0.500 (SD = 0.127), *M*_control_ = 0.484 (SD = 0.131); *t*_(46)_= 0.429, *p* = 0.483, cohen’d = 0.12) or mean error (*M*_experimental_ = 0.097 (SD = 0.035), *M*_control_ = 0.098 (SD = 0.031); *t*_(38)_ = −0.057, *p* = 0.973, cohen’d = 0.03) between the two groups. Meanwhile, independent sample *t*-test results indicated that there were no significant differences in one’s mastery goal orientation (*M*_experimental_ = 5.854 (SD = 0.773), *M*_control_ = 5.819 (SD = 0.832); *t*_(46)_= −0.150, *p* = 0.713, cohen’d = 0.04) or performance goal orientation (*M*_experimental_ = 5.063 (SD = 0.887), *M*_control_ = 5.146 (SD = 0.893); *t*_(46)_= 0.324, *p* = 0.673, cohen’d = 0.12) between the two groups.

### ERP Results

**Figure 2:**
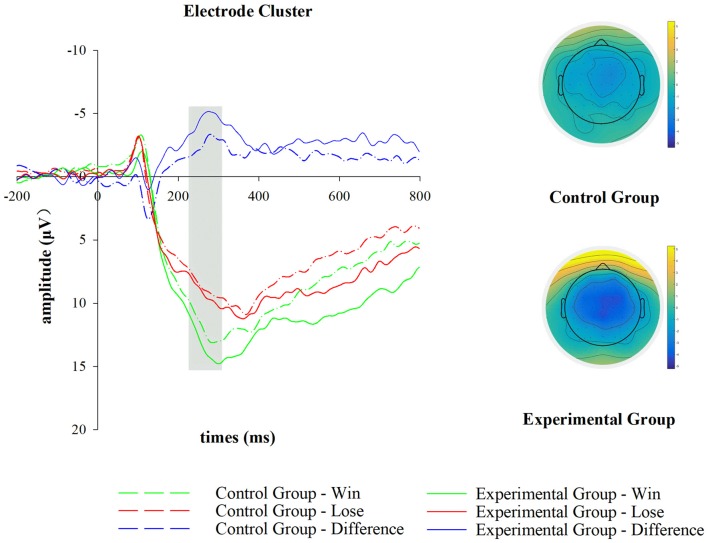
Feedback-related negativity (FRN) results during the outcome appraisal stage. For illustration, grand-averaged event-related potential (ERP) waveforms of FRN and FRN loss-win difference wave (d-FRN) from the electrode cluster (FC1, FCz, FC2) are shown for group (control group versus experimental group) and outcome (win versus lose) conditions. The scalp topographic distribution of d-FRN is provided for control and experimental groups, and the bar for the topographic map ranges from −5 μV to 5 μV.

After EEG data processing, the averaged trial numbers are *M*_win_ = 27.62 (SD_win_ = 7.44) and *M*_lose_ = 26.71 (SD_lose_ = 6.78) in the control group, while are *M*_win_ = 28.52 (SD_win_ = 8.88) and *M*_lose_ = 27.42 (SD_lose_ = 8.49) in the experimental group, which are comparable to each other. As demonstrated in Figure 2, the mean FRN amplitudes were 12.998 μV (experimental group-win), 8.592 μV (experimental group-lose), 10.608 μV (control group-win) and 8.492 μV (control group-lose) in respective conditions. An ANOVA analysis for the FRN showed a significant main effect of outcome (*F*_(1,46)_ = 50.605; *p* < 0.001; *η*^2^ = 0.524). However, the main effect of group was not significant (*F*_(1,46)_ = 0.614; *p* = 0.437; *η*^2^ = 0.013). The main effect of outcome indicated that there was a more negative FRN in the losing condition than in the winning condition. In addition, the significant interaction effect between group and outcome (*F*_(1,46)_ = 6.243; *p* = 0.016; *η*^2^ = 0.119) indicated that the d-FRN amplitude in the experimental group (−4.406 μV) was more pronounced compared with that in the control group (−2.115 μV). Because of the significant interaction effect between outcome and group, simple effect analyses were subsequently conducted. Negative feedback was found to elicit a more negative deflection than the positive one in both experimental (*F*_(1,23)_ = 37.645; *p* < 0.001; *η*^2^ = 0.621) and control groups (*F*_(1,23)_ = 13.781.538; *p* < 0.01; *η*^2^ = 0.375). Meanwhile, no significant between-group differences were observed either when positive feedback (*F*_(1,46)_ = 1.855; *p* = 0.18; *η*^2^ = 0.039) or negative feedback (*F*_(1,46)_ = 0.004; *p* = 0.949; *η*^2^ = 0.001) was provided.

Combining behavioral and electrophysiological data, we found that one’s competence frustration negatively correlated with the mean d-FRN amplitude (*r* = −0.312, *p* = 0.031), while one’s performance goal orientation positively correlated with the mean d-FRN amplitude (*r* = 0.457, *p* < 0.01). To be specific, while performance goal orientation significantly correlated with the mean d-FRN amplitude in the experimental group (*r* = 0.591, *p* < 0.01), we did not find such a relationship in participants of the control group (*r* = 0.326, *p* = 0.120). There was no significant correlation between one’s mastery goal orientation and the mean d-FRN amplitude (*r* = 0.020, *p* = 0.895) in this study.

## Discussion

### The Restoration Process of Frustrated Competence

According to SDT, the satisfaction of each basic psychological need is fundamental for the maintenance of one’s optimal functioning and well-being (Deci and Ryan, 2000). Recent evidences suggested that, in response to need frustration, individuals may take active actions to restore it through self-regulation (Fiske, 2004; Veltkamp et al., 2009). In line with this reasoning, a number of studies conducted by Radel et al. (2011, 2013, 2014) have explored the effect of prior autonomy frustration on one’s motivation, attention and decision-making in a subsequent activity if their perceived autonomy can get restored in it. Moreover, a few pioneering studies have explored the relatedness restoration process and the effect of prior relatedness frustration on one’s subsequent behaviors (Gardner et al., 2000; Pickett et al., 2004; DeWall et al., 2008). As a comparison, few (if any) studies have examined the restoration process of competence. To fill this research gap, in a recent field study conducted in the educational setting, we revealed a potentially positive effect of competence frustration outside of its primary thwarting context (Fang et al., 2017). Extending our previous study, we adopted a between-subjects design and directly manipulated one’ competence frustration in this experimental study. The EEGs of our participants were recorded all along, which makes it possible for us to examine the effect of prior competence frustration on one’s motivation to win in a subsequent competence-supportive activity.

This experiment consisted of two sessions, and participants in both the experimental group and the control group were instructed to work on the TE task in session 1 and the SW task in session 2. According to the theoretical reasoning of the need restoration process, once a basic psychological need gets frustrated, one would actively take part in another activity and get immersed in it if this activity can restore his/her frustrated need (Radel et al., 2013). It is fundamental for the second activity to be different from the original one, as it would be very difficult for one to restore their frustrated need in the same activity, even if this activity becomes more need-supportive than before (Radel et al., 2013). Accordingly, to create an opportunity for competence restoration, we adopted different tasks in different sessions. In this study, competence frustration was manipulated through task difficulty. Thus, while participants in both groups worked on the TE task in session 1, participants in the experimental group were faced with an overwhelmingly difficult TE task. To give them the opportunity to restore their competence in session 2, the SW task was set as moderately difficult, which is competence-supportive (Ma et al., 2017). As a control, those in the control group worked on moderately difficult tasks in both sessions.

Previous literatures consistently showed that the FRN loomed larger in response to the negative feedback compared with the positive one (San Martín, 2012). Accordingly, we found the valence effect on the magnitude of the FRN in both the experimental and the control group. The key finding of this study is that participants in the experimental group showed an enlarged d-FRN toward feedback outcomes compared with those in the control group. In the pioneering study that proposed the motivational significance account of FRN, Gehring applied a binary choice gambling task in which subjects were asked to choose between 5 and 25, which would lead to either a gain or a loss of the corresponding amount of money. When choosing 25, outcomes of the gambling task bear more motivational significance to the participants. A larger d-FRN was observed when participants chose 25 instead of 5. Based on this discovery, Gehring and Willoughby (2002) argued that the amplitude of d-FRN may reflect one’s motivation level in terms of outcome evaluation. Similar findings were reported in effort-requiring tasks, as the mere confirmative action (Zhou et al., 2010), the additional effort put into a task (Ma et al., 2014b), as well as the opportunity to choose between equally difficult tasks (Meng and Ma, 2015) all resulted in a greater motivation to win and contributed to the enhanced d-FRN upon feedback. To sum up, a growing number of studies have demonstrated that FRN is a reflection of the motivational impact on the processing of outcome stimuli (Gehring and Willoughby, 2002; Yeung et al., 2005; Masaki et al., 2006; Zhou et al., 2010). In the current study, we observed a more pronounced d-FRN in the experimental group during session 2. In line with the motivational significance theory of FRN, this finding suggested that prior competence frustration strengthened one’s motivation to win in a subsequent less-demanding task, which provided empirical evidence for the competence restoration process.

### d-FRN as a Tentative Neural Indicator of Intrinsic Motivation

Intrinsic motivation refers to one’s spontaneous potential to be curious and interested, to look for challenges and cultivate their skills and knowledge in the absence of external rewards (Deci and Ryan, 2000). In recent years, a number of pioneering studies have explored the neural underpinnings of intrinsic motivation (Murayama et al., 2010; Albrecht et al., 2014; DePasque and Tricomi, 2015; Marsden et al., 2015; Meng and Ma, 2015). According to recent literature reviews on the progress of neuroscientific investigations of intrinsic motivation, when participating in intrinsically motivated activities, individuals’ dopaminergic value system would be responsive to cues that signal task-related progress (Di Domenico and Ryan, 2017; Reeve and Lee, 2018). To be specific, as the anterior striatum has been well established to be responsible for the processing of feedback information (Tricomi et al., 2006; DePasque and Tricomi, 2015), most researchers who applied the functional magnetic resonance imaging (fMRI) technique resorted to the blood oxygen-level dependent (BOLD) signal in the anterior striatum during outcome evaluation to measure one’ intrinsic motivation (Murayama et al., 2010; DePasque and Tricomi, 2015). In a similar manner, researchers who adopted an electrophysiological approach focused on one’s neural responses to success and failure feedbacks in effort tasks and adopted the d-FRN to measure one’s intrinsic motivation (Ma et al., 2014a; Meng and Ma, 2015).

In the first electrophysiological study that examined intrinsic motivation, the researchers modified the experimental paradigm of Murayama et al. (2010) to explore the crowding out effect of monetary incentives on one’s intrinsic motivation (Ma et al., 2014a). They discovered that the d-FRN toward inherent lose-win divergence was significantly reduced if extrinsic rewards were once given but no longer available in the experimental group. However, this phenomenon was not observed in the control group (Ma et al., 2014a). In another study that explored the relationship between autonomy satisfaction and intrinsic motivation, the researchers manipulated the opportunity to choose between equally difficult tasks, and participants received a fixed payment irrelevant to their task performances. It was found that satisfaction of autonomy through the provision of choices brought a prominently more negative d-FRN toward performance feedback (Meng and Ma, 2015). In these two studies, performance feedback is unrelated with monetary rewards, and participants are assumed to complete experimental tasks purely out of intrinsic motivation. As the motivational significance theory indicated the magnitude of d-FRN to reflect one’s motivation level, the researchers went a step further to suggest d-FRN as a candidate neural indicator of intrinsic motivation (Ma et al., 2014a; Meng and Ma, 2015).

In our experiment, subjects received a fixed payment irrelated with task performances. We observed a more pronounced d-FRN in the experimental group compared with the control group during session 2. In support of this group-level finding, we also found that competence frustration negatively correlated with the d-FRN amplitude in session 2. If d-FRN can be regarded as a neural index of intrinsic motivation, these findings would help establish the causal relationship between prior competence frustration and one’s strengthened intrinsic motivation in another competence-supportive activity. When conducting this study, we did not ask participants to rate their intrinsic motivation and perceived competence in session 2. A major reason is that we did not want to make our research content explicit to the participants. If they realized what we were trying to examine when filling the scales, their responses to the items might be biased. At present, as self-reported intrinsic motivation data supportive of the electrophysiological findings were not collected, we refrain from being conclusive and suggest this mechanism to be only a speculation. This might be a limitation of this study, and follow-up studies are highly welcome.

## Implications and Future Directions

Findings of this study contribute to the need restoration hypothesis built on SDT, according to which the frustration of basic psychological needs would lead to a restoration process (Fiske, 2004; Veltkamp et al., 2009). To date, studies have explored some need restoration processes activated by need frustration. For instance, converging evidences showed that individuals would take actions to regain relatedness by becoming more attentive to social information (Gardner et al., 2000), nonverbal social cues (Pickett et al., 2004) and signs of acceptance (DeWall et al., 2008) after experiencing relatedness frustration. Moreover, going through relatedness frustration increased one’s motivation to renew affiliative bonds with others (DeWall et al., 2008). Besides autonomy and relatedness, the existence of a restoration process of competence has been recently tested as well. In a pioneering field study conducted in an educational setting, we found that for students who had been competence-frustrated to a great extent in a preceding course, a restoration process would be activated if the current course can help restore their competence, as they showed enhanced intrinsic motivation in the current course (Fang et al., 2017). While findings of our previous field study are illuminating, we cannot establish a causal relationship between one’s competence frustration in a previous activity and need-restorative behaviors in the current activity. In this study, competence frustration was directly manipulated, which is among the very first experimental studies that directly examine the competence need restoration process. Our results further confirmed the need restoration hypothesis, as competence-frustrated participants were found to restore their competence through enhancing the motivation to win in a subsequent less-demanding task.

It is worth noting that, in this study, one’s behavioral response toward competence frustration was only examined during its early stage. Thus, whether a similar restoration process will still be activated once an individual endure consistent competence frustration remains to be examined. Previous studies on relatedness frustration suggested that if one remained relatedness-frustrated for a long period of time and did not have the opportunity to restore relatedness, they may compensate by becoming more aggressive (Twenge et al., 2001), less altruistic toward others (Twenge et al., 2007), or accepting passivity and perceiving worthlessness (Williams, 2009). While our results suggested that prior competence frustration may affect one’s motivation to win in subsequent competence-supportive activities, it is possible that one may not get the opportunity to restore competence in the short run and certain detrimental effects of competence frustration may last long. Thus, follow-up studies may consider examining the consequences of long-term suffering of competence frustration. The evolution of one’s need restoration strategy and behavioral responses over time may also be explored in future studies.

Another theoretical contribution of this study is that we provided preliminary electrophysiological evidence for achievement goal orientation theory. In our study, results from correlation analyses between personality traits and the d-FRN observed in session 2 showed that one’s performance goal orientation negatively correlated with the motivation to win (as a more negative d-FRN has been proposed to suggest an enhanced motivation to win). This finding held true for the experimental group only. Thus, among the participants whose competence got frustrated, those who normally care about task performances rather than task mastery would pay less attention to task performances and show attenuated motivation afterwards. This finding suggested that following competence frustration, performance goal orientation may impact one’s motivation level in a subsequent less demanding activity. In other words, while the competence restoration process might be common, there are individual differences concerning its intensity. If d-FRN can be seen as a neural indicator of intrinsic motivation, then results of this study are consistent with some theorists’ statement that the pursuit of performance goals has a negative effect on intrinsic motivation (Ryan et al., 1991; Kaplan and Maehr, 2007; Lee, 2010; Barić et al., 2014). Interestingly, we did not find a significant correlation between mastery goal orientation and the mean amplitude of d-FRN in session 2.

Findings of this study also provide important guidelines for the managerial practice. To begin with, as we found that individuals would take active actions to restore their competence after it had been frustrated, managers of enterprises should endeavor to protect competence of their employees, which is fundamental to their overall well-being. For instance, when the work is too demanding or challenging, managers may pay attention to giving timely positive feedback or providing moderate autonomy to the employees (Meng and Ma, 2015; Meng and Yang, 2018). Our findings also bear practical implications for work arrangement. In this study, we found a surprising source of self-motivation, as competence-frustrated individuals would activate a need-restorative process and show enhanced motivation in a subsequent competence-supportive activity. This is not to suggest that managers should deliberately undermine the perceived competence of their employees so as to motivate them later on. After all, according to predictions of SDT, one’s motivation would be threatened in the activity which frustrated their competence (Deci and Ryan, 2000; Ryan and Deci, 2017). Rather, we urge the managers to take advantage of the need restoration process. In the workplace, some work is inevitably demanding, and it is highly likely that competence of some employees would get frustrated. If this already happened, managers should try to guarantee that this work is to be followed by a comparatively simple one. This arrangement gives employees the opportunity to regain competence, and they would get immersed in their jobs. To conclude, reasonable work arrangement can intrigue employees’ work motivation and thus boost enterprise performances.

## Conclusion

In an experimental study, we manipulated task difficulty to explore the effect of prior competence frustration on one’s motivation to win in another competence-supportive activity. Electrophysiological evidences suggested that participants who experienced competence frustration beforehand would increase their motivation to win (as reflected in the magnitude of d-FRN) in a subsequent task if it could help restore competence, which provided direct empirical evidences for the competence restoration hypothesis built on SDT and important guidelines for the managerial practice. Thus, by examining effects of competence frustration outside of its primary thwarting context, we complement and extend existing findings on the dynamics between need frustration and one’s (intrinsic) motivation.

## Author Contributions

LM and HF conceived and designed the study. HF collected and analyzed the data. HF and LM interpreted the data and drafted the manuscript. LM, HF, BH, HjF, HjZ and ZM reviewed and edited the manuscript. LM administered the project.

## Conflict of Interest Statement

The authors declare that the research was conducted in the absence of any commercial or financial relationships that could be construed as a potential conflict of interest.
