# Supplementary material for: A Surprising Source of Self-Motivation: Prior Competence Frustration Strengthens One’s Motivation to Win in Another Competence-Supportive Activity
Source: Front Hum Neurosci. 2018 Aug 3;12:314. doi: 10.3389/fnhum.2018.00314 (PMC6085490; doi:10.3389/fnhum.2018.00314)
Supplement: Supplementary file 1 [file Presentation_1.pdf]

Supplementary Material

A Surprising Source of Self-Motivation: Prior Competence Frustration Strengthens One's Motivation to Win in Another Competence-supportive Activity

Hui Fang, Bin He, Huijian Fu, Huijun Zhang, Zan Mo, Liang Meng *

*** Correspondence:** Liang Meng (Shanghai International Studies University):

promise_land@zju.edu.cn; promise_land@shisu.edu.cn

1 Supplementary Tables

1.1 Supplementary Table 1

TABLE 1 | A Questionnaire about the Experimental Experience

This survey is carried out to learn about your perceptions about the time-estimation task you worked on in the first session. Thank you for your support and wish you all the best in your future endeavors.

Please draw a "circle" or put a "tick" on the number that best represents your feelings toward the time-estimation task you worked on in the first session.

Items	1: Do not fully agree			⟶			7: Totally agree
1. I have serious doubts whether I can play the time-estimation game well.	1	2	3	4	5	6	7
2. I feel disappointed with my performance in the time-estimation game.	1	2	3	4	5	6	7
3. I feel insecure about my abilities of playing the time-estimation game.	1	2	3	4	5	6	7
4. When I am playing the time-estimation game, I feel like a failure because of the mistakes I make.	1	2	3	4	5	6	7

1.2 Supplementary Table 2

TABLE 2 | A Questionnaire about Personality Traits

This questionnaire is for academic research purpose only. Your personal information will not be revealed to third parties. Thank you for your support and wish you all the best in your future endeavors.

Please draw a "circle" or put a "tick" on the number that best represents your opinions. Please fill in the questionnaire carefully and truthfully.

Items	1: Do not fully agree			⟶			7: Totally agree
1. I would rather do what I do well, and I don't want to do what I do badly.	1	2	3	4	5	6	7
2. It is important for me to do challenging work.	1	2	3	4	5	6	7
3. I feel very happy when I do things that I will not make any mistakes.	1	2	3	4	5	6	7
4. If I can't finish a task that is difficult for me, I will work harder next time.	1	2	3	4	5	6	7
5. My favorite thing is that I can do best.	1	2	3	4	5	6	7
6. I am willing to do the task which will help me to learn new things.	1	2	3	4	5	6	7
7. If I don't make any mistakes, I'll think I'm smart.	1	2	3	4	5	6	7
8. The opportunity to learn something new is important to me.	1	2	3	4	5	6	7
9. I like to do things that I did well previously.	1	2	3	4	5	6	7
10. Based on the previous experience, I will try my best to improve myself.	1	2	3	4	5	6	7
11. If I do something better than most of other people, I'll think I'm smart.	1	2	3	4	5	6	7
12. The opportunity to improve my ability is very important to me.	1	2	3	4	5	6	7
